# A Novel Credible Carbon Footprint Traceability System for Low Carbon Economy Using Blockchain Technology

**DOI:** 10.3390/ijerph191610316

**Published:** 2022-08-19

**Authors:** Chunhua Ju, Zhonghua Shen, Fuguang Bao, Pengtong Weng, Yihang Xu, Chonghuan Xu

**Affiliations:** 1 Department of Modern Business Research Center, Zhejiang Gongshang University, Hangzhou 310018, China; 2 School of Management Engineering and E-Business, Zhejiang Gongshang University, Hangzhou 310018, China; 3 Academy of Zhejiang Culture Industry Innovation and Development, Zhejiang Gongshang University, Hangzhou 310018, China; 4 School of Business Administration, Zhejiang Gongshang University, Hangzhou 310018, China

**Keywords:** carbon emission trading, carbon footprint, blockchain, IPFS, interactive traceability, traceability off the chain, type filtering

## Abstract

To achieve the goal of carbon neutrality, many countries have established regional carbon emission trading markets and tried to build a low-carbon economic system. At present, the implementation of carbon emission trading and low-carbon economic systems faces many challenges such as manipulation, corruption, opacity, lack of trust, and lack of data tracking means. The application of blockchain technology can perfectly solve the above problems. However, the data recorded on a blockchain are often multi-type and heterogeneous, and users at different levels such as regulators, enterprises, and consumers have different requirements for data types and granularity. This requires a quick and trustworthy method for monitoring the carbon footprint of enterprises and products. In this paper, the carbon footprint traceability of enterprises and products is taken as an application scenario, and the distributed traceability concept of “traceability off the chain and verification on the chain” is adopted. By reconstructing the pointer of the file structure of the distributed storage, an interactive traceability structure supporting type filtering is constructed, which enables fast retrieval and locating of carbon emission data in the mixed data on the chain. The experimental results show that using the interactive traceability structure that supports type filtering for traceability not only releases the computing power of full nodes but also greatly improves the traceability efficiency of the long-span transaction chain. The proposed carbon footprint traceability system can rapidly trace and track data on an enterprise’s and a product’s carbon footprint, as well as meet the needs of users at all levels for traceability. It also offers more advantages when handling large amounts of data requests.

## 1. Introduction

Along with economic growth, greenhouse gas emissions are increasing, and environmental issues are affecting every aspect of every country in the world [1,2]. Greenhouse gas emissions exacerbate climate change and can severely impact the environment [3]. Therefore, there is an urgent need to control human activities to reduce the effects of greenhouse gas emissions on the environment. An emission trading system (ETS) is one of these proposed solutions. The ETS, performing as an authorized center, formulates a target of carbon emission reduction to manage the carbon emission permit (CEP) trading [4]. Zhang et al. [5] and Dong et al. [6] showed that carbon emission trading policies can effectively reduce carbon emission in pilot cities and that a carbon emission trading system is essential to achieve the goal of reducing carbon emissions. However, the current implementation of carbon emission trading faces many challenges, including the following: (1) the calculation of carbon budgets is prone to manipulation and abuse; (2) the carbon emission trading market is corrupt and opaque; (3) the process of allocating CEP is complex; and (4) carbon emission trading lacks tracking measurements [7]. By using blockchain technology, carbon footprints can be established on the distributed super ledger, forming a carbon footprint tracking system. This tracking system is necessary to eliminate corruption and to ensure that the records are kept unchanged to prevent manipulation [8,9,10,11]. Therefore, to fulfill the aforementioned system requirements, a technical architecture that can be used for carbon resource management and traceability is required. Blockchain technology represents a good solution, which can guarantee the transparency of the entire supply chain and make the data on carbon emission and carbon footprints “trustworthy”. With transparent and credible data, enterprises can carry out effective carbon disclosure and formulate corresponding carbon compensation schemes, to realize the real carbon neutral supply chain and negative carbon supply chain. Using blockchain and privacy computing technology, information can be verified but invisible, and all quantifiable carbon emission data can be safely shared [12]. Once the concerns about enterprise data security are eliminated, carbon emission data can be circulated and shared at the industrial level, and the value of the data can be truly utilized, which will promote the process of achieving the goal of carbon neutrality. Blockchain is favored in the field of carbon emission due to the frequent occurrence of carbon emission data falsification. In the background of addressing global climate issues, companies and brands not only need to comply with various “green agreements” and regularly report carbon emission data but also need to provide valid proof for the authenticity of these data. In recent years, the problems of “carbon emission data falsification” and “illegal carbon trading” in the low-carbon market have been revealed frequently, and the question concerning how to ensure the transparency and openness of carbon footprint and avoid being cheated is a big problem that people have been studying but not yet been able to solve [13]. Blockchain technology brings new hope for carbon footprint traceability since it is transparent, tamper-evident, and traceable.

Regarding the application of blockchain technology in complex application scenarios, we have developed an interactive traceability structure for agricultural products and realized the traceability solution of off-chain traceability and on-chain verification [14]. Due to the adoption of blockchain-based distributed data storage and the decentralized traceability process, the storage structure has significant advantages in terms of tamper resistance, transparency, and traceability efficiency. It is very suitable for fast traceability and real-time monitoring of peer-to-peer transactions in networks lacking trust. However, this system only supports “full-chain and full-information” traceability, which means that each traceability will return all data of the entire transaction chain and, therefore, will return much information unrelated to carbon emission when providing supporting documents for carbon emission trading. In this paper, we propose to improve the interactive traceability structure mentioned above and adopt the distributed traceability concept of “off-chain traceability and on-chain verification”. In this paper, to satisfy the demand for regulation, tracking, and tracing of different types of data from different levels of users, such as regulators, producers, and consumers in the carbon emission trading market, an interactive traceability structure supporting type filtering is built to support the tracing and tracking carbon emission-related information in a transaction chain. It enables one to address the problems of manipulation, corruption, opacity, and lack of data tracking tools in the carbon emission trading system. At the same time, it adopts the concept of a decentralized traceability process and optimizes the distributed storage structure in the traceability system so that the whole system can not only cope with ultra-high-amounts traceability requests but also efficiently trace the carbon emission-related data. It realizes the rapid traceability and tracking of enterprise and product carbon footprint data.

**Figure 1:**
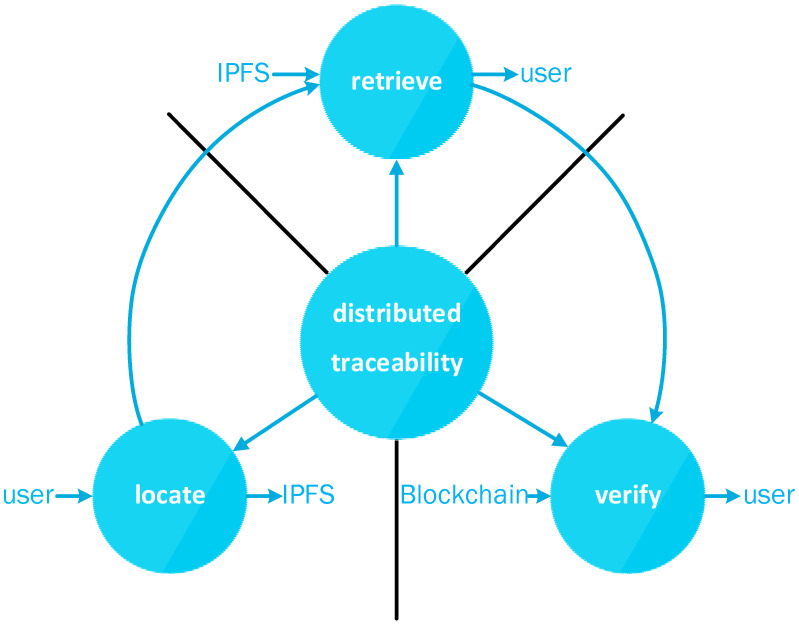
This paper subdivides the distributed traceability process into three stages: locating, retrieving, and verifying.

As shown in Figure 1, this paper divides the distributed traceability process into three stages: locating, retrieving, and verifying. We believe that traceability should be separated from verification, that the consensus and verification function of blockchain should be strengthened, and that the remaining work should be handed over to a more suitable distributed storage system (e.g., IPFS). In addition, this paper further refines the steps in the traceability process and further decomposes the traceability process into locating and retrieving so that the locating and retrieving processes do not interfere with each other, thus making it possible for the locating and retrieval to be parallel and reducing the impact of document filtering operations on the traceability efficiency. In this paper, the steps and functions are separated by redesigning the file structure of distributed storage and the tracing process. By further optimizing the storage structure, a three-level structure is adopted, and the pointer structure is used as a separate layer to store the locating information so that the pointer layer corresponds to the positioning function alone. Firstly, the corresponding information is located in the pointer level, and then those files are selected to obtain it, thus realizing the traceability with simple semantic definition. Compared with the previous methods of staggered locating and retrieval in distributed traceability, the traceability method in this paper not only ensures the traceability efficiency but also provides strong flexibility, making it possible to filter distributed storage data efficiently.

In Section 3, this paper specifies the design of the interactive traceability structure and traceability process of the carbon footprint traceability system. In Section 3.1, we will first explain how the special storage structure design is carried out in this paper to build the interactive traceability structure supporting type filtering. This section will explain how data are stored in the blockchain and IPFS systems and how the interrelationship between transactions, pointers files, and evidence is established. Afterward, in Section 3.2, we elaborate on how to perform traceability on the distributed storage structure of Section 3.1. In Section 4, we use simulation experiments to verify the feasibility and traceability efficiency of the carbon footprint traceability system proposed in this paper.

## 2. Literature Review

In this part, we first outlined the research and application status of blockchain technology in carbon trading, energy trading, and carbon footprint, and emphasized the importance of different needs at different levels of participants in carbon trading and carbon footprint scenarios, as well as the shortcomings of blockchain technology in practical applications. To solve these problems, we put forward the technical idea of “Blockchain + IPFS” and the concept of distributed traceability, making a detailed summary of research in related fields. At the same time, the problems encountered in the practical application of blockchain + IPFS technology and the concept of distributed traceability are expounded, and solutions to these problems are given.

The unique features of blockchain, such as security, invariance, transparency, traceability, and trust, make it a robust and reliable solution for the carbon emission trading market, and it can be well applied to traceability systems. Empowered by blockchain and smart contracts, a tamperproof trust system can be constructed to track data and be able to share records with authorized organizations, giving them the ability to track carbon footprint issues throughout the supply chain [15,16]. In agriculture, minerals, and energy, Guillaume Chapron, and Hua Weiqi applied blockchain technology to supply chain traceability and energy trading [17,18]. In China’s energy sector, the applications of blockchain are just beginning, and some practical explorations are already underway in the fields of power grids, new energy, and carbon emission reduction. The Carbon Tracker Platform (COT), designed to track carbon emission from “mining to the final product”, has completed validation of the concept and continues to evolve since its initial launch in October 2019 [19]. Alia Sadavi et al. [7], Fang Yuan Zhao et al. [20], Weiqi Hua et al. [18], Minglin Sun et al. [21], Michael Wang et al. [15], and Zhuo Hu et al. [4] have also made relevant research and exploration on the application of blockchain technology in the field of carbon trading. In the field of carbon trading, most articles build the system from the government’s perspective, so as to facilitate the government’s supervision of the carbon trading market, enhance the transparency and credibility of the carbon trading market, and thus put an end to corruption and manipulation. However, Hua Weiqi et al. [18] and Michael Wang et al. [15] studied issues from the perspective of consumers and enterprises, emphasized the influence of consumers’ and enterprises’ behaviors on the whole system, and thus concluded that the construction of a carbon trading system should pay attention to the different needs of more participating parties. Davit Marikyan et al. [22] studied the impact of user subjective factors on the implementation of blockchain system. The design of the system should pay attention to users’ preferences and knowledge level, and provide users with more images, videos, billing documents, and other data with strong presentation. Applying blockchain technology for product traceability can help foster market trust in products and brands, as well as give customers a better understanding of how products are made, and likewise create a better customer experience, allowing consumers to gain insight into how their strategic choices impact the environment [23]. There are many advantages of blockchain technology in energy trading, carbon emission trading, and the low-carbon products trading market, which solve the trust problem in the peer-to-peer trading system, match the trading demand between users, and optimize energy allocation. These advantages are significant in international trading, thus promoting the international trading of energy. Consumers are usually open to the application of new technologies, which is both an opportunity and a challenge for all parties in the supply chain, manufacturing, and circulation of products. Usually, they are concerned about the risk of compromise risk in this process of data management process, and the cost of application is also a factor to be considered [24]. Also, the problems of using blockchain alone to store data are apparent, the information that can be stored on the chain is limited; the level of applicability for complex scenarios is low, and timely traceability queries are complicated under a large number of accesses; the throughput capacity of information is poor, etc. [14,25,26,27].

One of the solutions to the above problem is to use blockchain technology in combination with IPFS (Inter Planetary File System). In the research direction of data storage and protection, many researchers have proposed solutions that combine blockchain and IPFS for their respective research fields. Wei Ren et al. [28], MING LI et al. [29] used blockchain + IPFS to store unstructured data, so as to enhance the security and trustworthiness of data, including agricultural sampling data protection for IoT networks and image big data protection based on compressed sensing. Jieren Cheng et al. [30], Chin-Ling Chen et al. [31] and have proposed applications of blockchain + IPFS systems from the perspective of data storage and transmission, including application scenarios such as collaborative DDoS attack detection, anonymization protocols, and secure data transmission schemes. In the research direction of enhancing the credibility of deposition, the research of Randhir Kumar et al. [32], Jin Sun et al. [33], Shaojing Fu et al. [34], and Moses Arhinful Acquah et al. [35] use the advantages of blockchain + IPFS technology to solve the existing trust problem of data deposition. Their research covers copyright protection of image and video sharing system, secure storage and efficient sharing of electronic medical records, verification of data retrieval and file integrity, and protection of encrypted fingerprint identification data through symmetric encryption. Owen Lo et al. [36], Hyoeun Ye, and Sejin Park et al. [37] propose the use of blockchain + IPFS technology to enhance the overall efficiency of the system, and their research covers the use of blockchain+ IPFS in the field of e-governance and vehicle data storage. There are also concerns that the application of blockchain technology to traceability systems will lead to higher energy consumption similar to that of bitcoin mining [38]. In the traceability system required by the carbon trading market, we adopt the federal chain structure to ensure that all nodes with certified transactions are owned by the parties in the process of production and circulation of products, and no private nodes has the right to disturb the information on the chain. Additionally, we can also take a special design so that the ultra-high-volume traceability queries from consumers or market regulators can be transferred outside the blockchain to avoid congestion and high energy consumption. Facts have proved that the combination of blockchain technology and IPFS meets people’s expectations at a reasonable cost.

It is obvious that with the combination of IPFS technology, the storage problem of large volumes, and unstructured data has been resolved, which significantly extends the application scenarios of blockchain technology. However, at the same time, many different types of heterogeneous data on the chain also pose a significant problem for data retrieval and access. Jin Sun et al. [33], Shaojing Fu et al. [34], Owen Lo et al. [36], and Sejin Park et al. [37] discussed the data retrieval and access efficiency in distributed storage environment, and their research is mainly aimed at the retrieval of a single file. These retrieval methods will cause great pressure on the full node in a large-scale market trading system. In a large-scale trading system without enough trust, both supervision and trading behavior require a lot of proof data to support it, so the question concerning how to quickly obtain specific data as proof is a problem. The requirements of different user levels in the traceability system are also different [39,40,41]. This system mainly needs to meet the market supervision of government agencies, carbon emission trading at the enterprise level, and the traceability and anti-counterfeiting requirements at the consumer level. This requires that the traceability system carry out different data types and granularity of traceability.

This paper constructs a carbon footprint traceability system for regulators, producers, and consumers in order to meet the carbon footprint traceability or verification requirements at different levels in the carbon emission trading scenario, and provide a credible enterprise carbon footprint of enterprises and products. Based on the concept of distributing traceability, we reconstructed the storage structure of distributed files and established a new interactive traceability process supporting type filtering. It realizes the subdivision of the locating, retrieving, and verifying processes in distributed tracing, and enables the carbon footprint traceability system to retrieve and verify the limited and filtered data after locating. The application of this system can speed up the tracking of carbon emission records in various production stages of enterprises, ensure the accuracy of the data, and facilitate consumers, third-party verification agencies, and departments responsible for carbon emission management to verify and trace the emissions.

## 3. Interaction Structure Design of Carbon Footprint Traceability System

In the carbon emission trading and low carbon market, regulators, producers, and consumers mostly focus on specific data and evidence related to carbon emission, while the information recorded in the blockchain often includes various types and forms of information, which leads to inefficient screening and tracing of carbon emission-related information. In this paper, to cope with the problem of returning a large amount of non-critical information when tracing the source, we propose a method that can trace specific categories of data, allowing us to quickly locate historical data related to carbon emission. In this paper, by adding pointers to distributed storage files, a specific file structure is formed for traceability. The traceability process is separated from the verification process, therefore realizing the distributed traceability of “traceability off the chain and verification on the chain” [14]. The traceability structure and process we proposed in the previous study returns all of the data of the transaction chain, which leads to inefficient traceability of specific types of data when the on-chain data volume is huge and the types are too diverse. When it is applied to the carbon footprint traceability scenario, users will receive a large amount of data that is unrelated to carbon emission (even if these data are related to the transaction chain). Therefore, further improvements have been made in this paper, aiming to achieve rapid traceability and retrieval of specific data in the transaction chain, and form an interactive traceability structure that supports type filtering.

### 3.1. Interaction Structure Design

As analyzed in the literature review, the joint application of blockchain and IPFS could solve the storage problem of large volumes and unstructured data. Furthermore, it dramatically extends the application scenarios of blockchain technology. After further optimization of the distributed storage structure and traceability process, the structure proposed in the previous study can already cope with higher-volume data traceability requests, the efficiency of data traceability has been greatly improved, and it has realized the distributed traceability of “traceability off the chain and verification on the chain”, which greatly reduces the data retrieval pressure of full nodes. However, when the data types on the chain are complex and diverse in practical applications, and when users only need specific types of data to be verified, it is necessary to optimize further and improve the existing structure.

**Figure 2:**
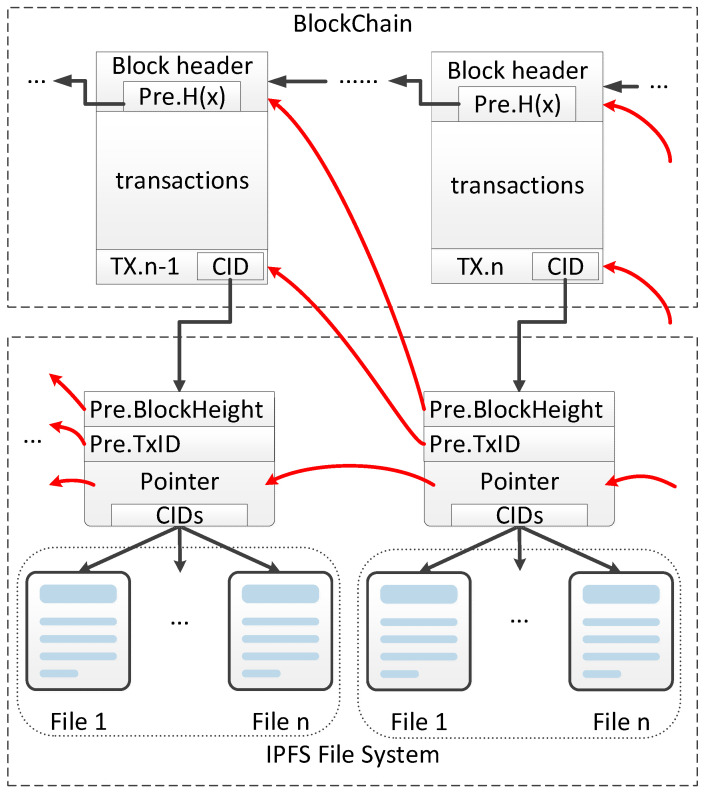
Pointer design.

In this paper, we adopt the solution idea of “storing validation and index data on the chain, and storing a large volume of raw data in IPFS” [28,29,33,34,37], and optimize the structure of the original evidence file based on the previous study. We classify the raw files in advance when they are deposited in IPFS, and the related classification information is stored separately from the related pointer information. The specific pointer design is shown in Figure 2.

Where Tx.n−1 and Tx.n are the corresponding transaction information contained in a certain two adjacent transactions Tx(Transaction) in the transaction chain, and we define Tx as

Tx = [Content, Author, Pre.TxID, CID, TxID]

Content means the text content of the transaction. Just as in a standard transaction, it records the transaction status information, account information, related verification information, etc.; and Author means the editor of the transaction information and the uploader of the related evidence, which generally refers to the initiator of the transaction. Pre.TxID is the hash of the previous transaction in the transaction chain; CID is a pointer to the IPFS system, pointing to the pointer file corresponding to this transaction, obtained after the Author classifies the deposit file and uploads the pointer file; TxID indicates the hash of this transaction, which must be calculated after obtaining the CID, with TxID = Hash (Content, Author, Pre.TxID, CID, TxID).

The pointer file in this paper is a JSON format file, and the composition of the pointer file content is

Pointer File = [Pre.BlockHeight, Pre.TxID, Pre.CID, Category, CIDs, Chain level]

Pre.BlockHeight is the height of the block in the previous phase of the transaction; Pre.TxID is the hash value of the previous phase of the transaction; Pre.CID is the hash value of the pointer file corresponding to the previous phase of the transaction; Category is the type of the corresponding evidence file, which is only divided into three types in this paper, C (correlation), I (indirect correlation), and N (no correlation), where C means the document is correlated with carbon emission, I means the document is indirectly correlated with carbon emission, and N means the document is not correlated with carbon emission. In the subsequent traceability process, the documents can be obtained by the type of deposited documents needed. CIDs are the set of CIDs of the corresponding depository files of this transaction, and their order should correspond to Category one by one; Chain level indicates the position of the transaction corresponding to the current pointer file on the transaction chain and is used to establish the correspondence between the pointer file and the transaction.

**Figure 3:**
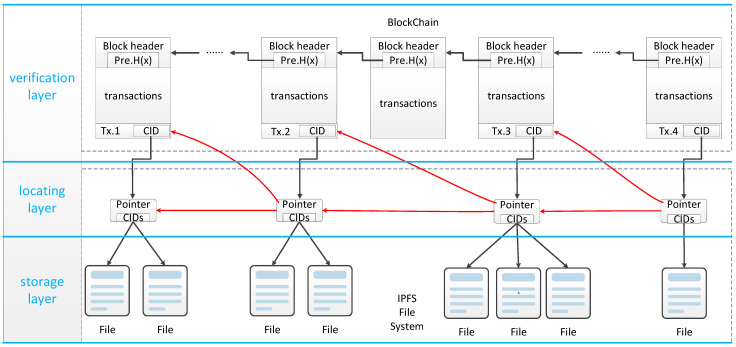
The three-layer interactive traceability structure supporting type filtering.

After the pointer design, the final interactive traceability structure supporting type filtering is shown in Figure 3.

From Figure 3, we can see that the interactive traceability structure supporting type filtering proposed in this section is generally divided into three layers. All transaction data in the market are recorded on the blockchain, but only the necessary index data and verification data are included. The original data related to transactions are stored in IPFS. The original data in IPFs and the transaction data on the blockchain are connected via a layer of pointer files. Through the unique CID pointer recorded in the transaction and the CIDS of the original data recorded in the pointer files, the data retrieval from the blockchain to the IPFs system can be realized. The pointer files also contain a pointer to a specific transaction in the blockchain, which is used to locate the appropriate block in the blockchain by going back from the files in IPFS. This bi-directional pointer structure between blockchain and IPFS provides more options for the traceability process. The traceability process can be completed not only in blockchain but also in IPFS. The corresponding pointer files are connected according to the relationship between the transaction, which makes it possible to trace the source directly in the file structure formed by the pointer files when we have the latest transaction information, and in this process, we can also accurately locate the corresponding information in the blockchain in reverse. This enables users to complete traceability in IPFS by themselves and then send authentication requests to the blockchain system.

### 3.2. Interactive Traceability Process

**Figure 4:**
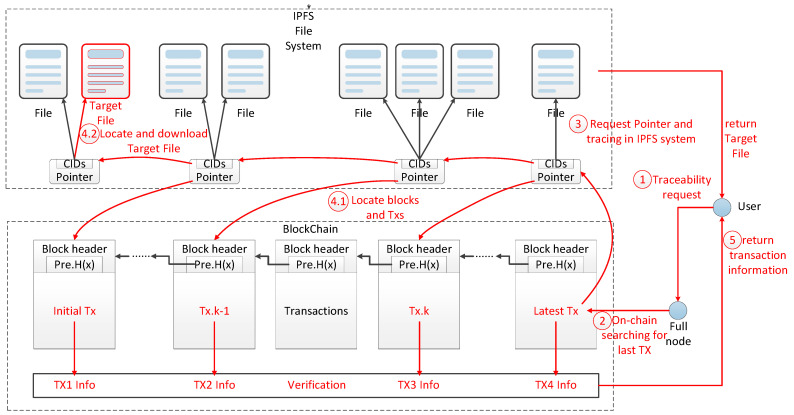
The traceability process under the interactive traceability structure supporting type filtering.

The Interactive traceability structure supporting type filtering present in this paper adopts the concept of “traceability off the chain and verification on the chain” [14]. The full node just needs to locate the most recent transaction information on the blockchain, submit a file acquisition request to the IPFS system based on the CID in the transaction information, and return the pointer file when it receives the user’s request for traceability. The user can then perform historical documentation traceability independently using the pointer file and obtain the historical pointer files as an index. The relevant evidence files may then be downloaded in accordance with the pointer file’s category information, and the verification request is submitted to the full node following the block information. The full node finds the transaction location following the request, obtains the relevant transaction data and verification data, and provides them to the user so that they may locally confirm the validity of the relevant transaction and the evidence files. Figure 4 demonstrates this traceability process. As shown in Figure 4, after the retrieval of the traceability process, only the Target File is related to carbon emission trading, so finally, only all of the pointer files, transaction chain information, verification data, and Target File are returned to the user, and the rest of the original data not related to carbon emission trading is filtered out.

The traceability process named Algorithm 1 is as follows:
**Algorithm 1** Tracing the source of a certain transaction**Require: Last TxID**//The TxID of a certain transaction is given by the user node.**1: block height, CID, transactions chain ← search_last_TX(Last TxID)**//After receiving the request, the storage node searches the local database for the most recent transaction in the blockchain and saves the height of the block, the CIDs, and the complete content of that transaction.**2: api ← ipfshttpclient. connect()**//Initiate the InterPlanetary File System**3: Pointer file ← api.cat(CID)**//Use CID to download the latest Pointer file, and send it to the user.**4: while True:**   **pre_height, pre_TxID, pre_CID, CIDs, Category**
**←**
**to_json(Pointer file)**   //Convert Pointer file to JSON format, and return pre-step information.   **block height, TxIDs**
**←**
**list.append(pre_height, pre_TxID)**   //Record the corresponding position on the chain   **index_list = [a for a, b in enumerate(Category) if b == C]**   //Locate files that match the Category type C.   **Files**
**←**
**return_files(index_list, CIDs)**   //Return relevant files to the user   **if pre_CID:**     **Pointer file**
**←**
**api.cat(pre_CID)**   **else:**     **break**     //Use pre_CID to download the pre-stage Pointer file, and send it to the user. If pre_CID is empty, then end the process.**5:transaction chain←search_content(block height, TxIDs)**//Obtain the complete content of the transactions in a specific block. The user can then confirm the content as usual after receiving the whole transaction chain from the storage node.

## 4. Simulation Experiment and Analysis

In this paper, we validate the traceability efficiency of the proposed interactive traceability structure supporting type filtering through simulation experiments. In Section 4.1, we will explain the environment in which the simulation experiments are run and how the simulation data used is composed. In Section 4.2, we will use the traceability process in Section 3.2 for traceability, compare it with the traditional on-chain tracing method, and analyze the results.

### 4.1. Experimental Design

**Figure 5:**
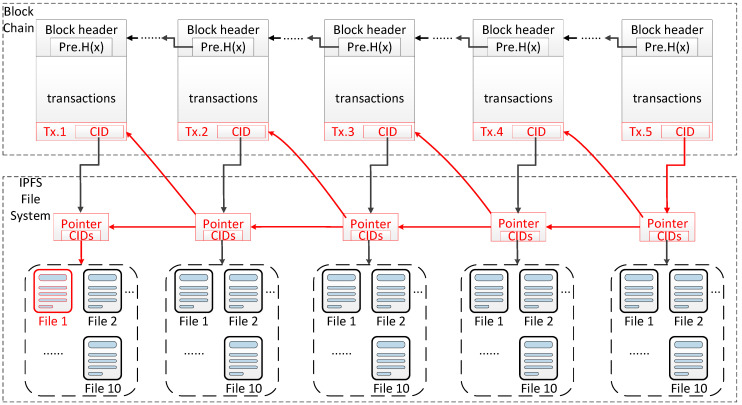
The interrelationship between transactions, pointers files, and evidence files in simulation.

The operating environment of the simulation experiment in this paper is as follows: the operating system is windows 10 (developed by Microsoft Corporation, Redmond, WA, USA), the memory size is 16 GB, the CPU is Intel(R) Core (TM) i7-10875H CPU @ 2.30 GHz, IPFS version 0.8.0 (developed by Protocol Labs, San Francisco, CA, USA), the network downstream is 28.9 Mbps, and the upstream is 29.19 Mbps. The structure of the transaction chain, the pointer file, and the corresponding deposition file are shown in Figure 5.

The blockchain simulation dataset is generated by local node simulation, and the traceability target transaction chain is a 5-level transaction chain with 10 evidence files for each transaction. That is, the latest on-chain transaction is Tx.5 at present, and in the condition that the TxID of Tx.5 is known, the carbon-related data information of the whole chain needs to be traced, and the original evidence files related to the carbon emission of the whole transaction chain information on the blockchain is requested to be returned. As shown in Figure 5, the first file of the first transaction in the simulation dataset is assumed to be related to carbon emission, which means that among the classification parameters recorded in its corresponding pointer file, there is Category = [C, N, N, N, N, N, N, N, N, N, N, N, N, N,]. Table 1 shows the contents of the pointer file in our set of simulation data.

As shown in Table 1, this pointer file corresponds to the 5th transaction and records the 10 evidence files of the 5th transaction. According to Category, it is known that all deposition files have nothing to do with carbon emission. In addition, it records that the previous transaction is in the 846th block, as well as the TxID of the previous transaction and the CID of its corresponding Pointer file.

### 4.2. Experimental Results and Analysis

**Figure 6:**
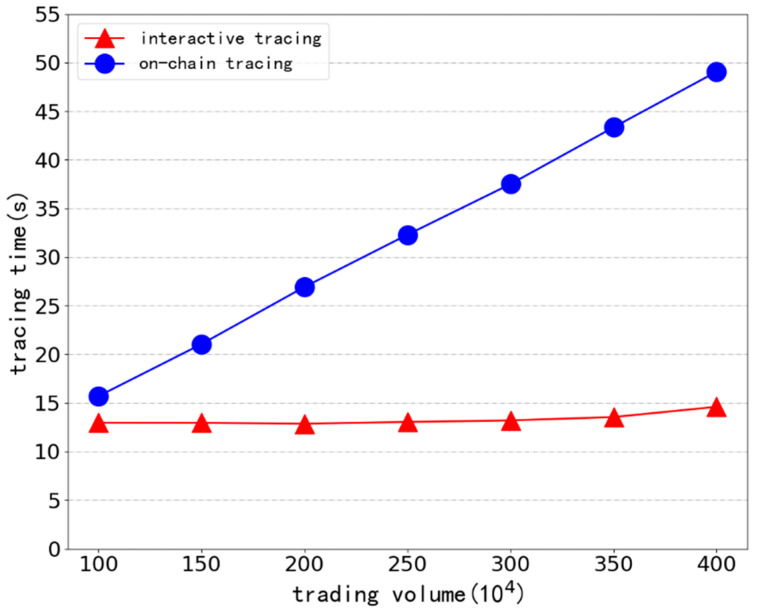
Comparison of the efficiency of interactive traceability structures supporting type filtering with traditional traceability.

To ensure the step-by-step increment of the span between the first and last transaction, we generate seven groups of simulation data with a total trading volume between 1 million and 4 million by local nodes, pack 200 transaction messages in each block, and randomly insert a target transaction in every fifth transaction volume. Different amounts of simulated data are used to vary the time between the first and last transactions, and the traceability impact of the interactive traceability structure is contrasted with that of the conventional on-chain traceability method. Figure 6 displays the overall amount of time spent on the associated algorithm tracing.

It can be seen that the total time spent by traditional on-chain traceability methods increases gradually as the transaction volume increases. When the total transaction volume reaches 4 million, the interactive traceability method takes only 14 s to retrieve the transaction chain with corresponding original documents and verification information to complete the traceability, while the traditional chain traceability takes more than three times as long (up to 49 s). The distributed traceability scheme proposed in this paper, which is based on the interactive traceability structure supporting type filtering and adopts the form of “traceability off the chain and verification on the chain“, maintains the same total traceability time and is unaffected by the span of transaction chains. The benefit of the interactive traceability structure is increasingly obvious the longer the block span is between the first and last transaction.

**Figure 7:**
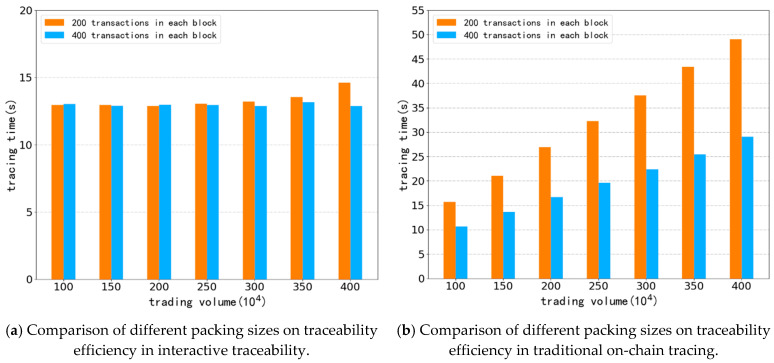
The influence of packaged transaction volume in each block on traceability efficiency.

**Figure 8:**
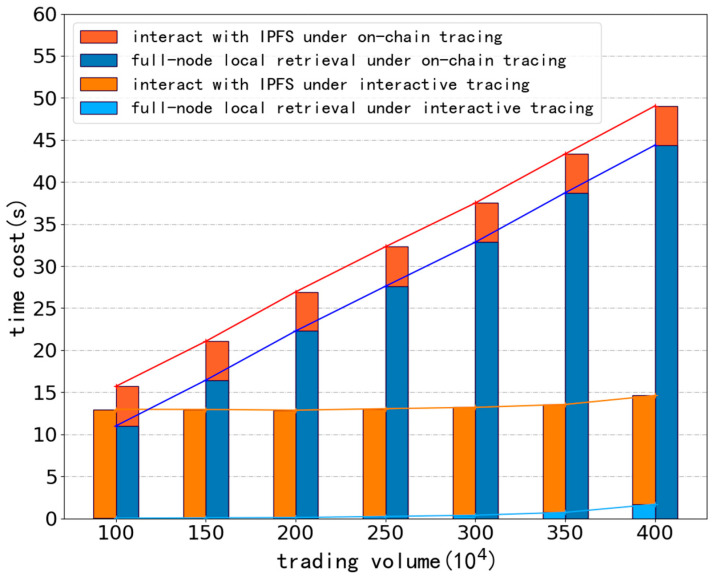
Comparison of traceability efficiency between two different traceability processes.

In our experiments, we also studied the impact of the number of transactions packaged in each block on the results. As shown in Figure 7, we used two simulation data sets for comparison; each block contains 200 transactions and 400 transactions. It can be seen in Figure 7a that the tracing efficiency in interactive traceability is hardly affected by the number of packaged transactions in each block, and the fluctuation of efficiency is mainly caused by changes of network conditions. This result is mainly due to the fact that the tracing process in our design is mainly carried out in the IPFS system, while the changes of parameters in the blockchain have little impact on tracing efficiency. In Figure 7b, we can see that when the number of packaged transactions in each block is doubled and there is a significant improvement in traceability efficiency in traditional on-chain traceability. This is due to the fac that, in on-chain traceability, the traceability process is mainly carried out on the blockchain, so the complexity of the blockchain directly affects traceability efficiency. However, in order to ensure the transmission efficiency in the peer-to-peer network, the number of packable transactions in a block is limited, so it is impractical to improve the traceability efficiency by continuously increasing the number of packets in a block. Figure 8 shows the traceability time consumption of two different traceability processes in the whole trial process, and also displays the weight of the blockchain system and the IPFS system in the traceability process.

Unlike in [14], it can be seen from Figure 8 that in the traditional on-chain tracing, the interaction time with IPFS is stable at about 4 s (mainly the time consumed when downloading the original file). Compared with the time spent interacting with IPFS, which has been stable at about 4 s, the time spent on local retrieval of traditional chain traceability is growing rapidly with the increase of the overall transaction amounts. When it is necessary to locate a transaction chain out of 4 million transactions, the full node will spend nearly 45 s searching locally. In the interactive traceability with type filtering support, the interaction with IPFS takes significantly more time than the traditional on-chain traceability approach. More than 90% of the time is spent by users interacting with the IPFS, which takes about 13 s. The main reason for this phenomenon is that the interactive traceability with type filtering support adds extra pointer files, which have to be downloaded before downloading the original evidence file. Although the size of the pointer file is negligible, the network latency will still have an effect.

Due to the concept of “off-chain traceability and on-chain verification”, the interactive traceability with support for type filtering allows the whole node to do almost no excessive local search, and the primary traceability process is carried out in the interaction between the user and IPFS, making it very advantageous when the span of the transaction chain is long.

### 4.3. Discussion

Judging from the experimental results, the carbon footprint traceability system proposed in this paper can perfectly achieve the task of traceability of the entire transaction chain and retrieval of carbon emission-related original data. Moreover, the distributed traceability concept of “traceability off the chain and verification on the chain“ is adopted, full nodes can free up more storage space and traceability computing power, thus making the work focus of authoritative nodes shift to transaction tracking supervision and verification, which is beneficial to reducing system errors and improving the overall system operation efficiency and robustness. The efficiency advantage of the carbon footprint traceability system proposed in this paper is significant, especially in the long transaction span.

**Figure 9:**
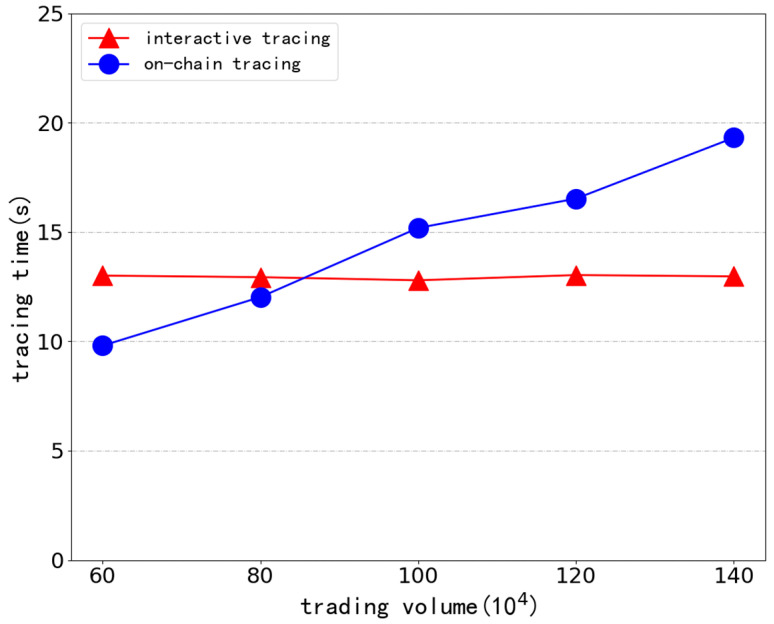
When the transaction chain span is too short, the traceability efficiency is lower than that of the traditional method.

But at the same time, this system has a stronger dependence on the Internet, so the network conditions have a more significant impact on its overall operating efficiency. As shown in Figure 9, in our simulation experiment, when the transaction chain span is less than 0.8 million transactions, the tracing efficiency is lower than that of the traditional method due to the network delay and bandwidth limitation.

However, assuming that 200 transactions are packaged per block, and a block is generated every 10 min, the time span is less than one month. Of course, in practice, due to the different choices of consensus algorithms, block generation frequency may be higher, and the transaction volume packaged in each block is more than in the experimental setting. Therefore, the actual time span may be about a week. When the traceability time span is higher than this, the efficiency advantage of the system begins to appear, and the advantage becomes more obvious as the span increases. Of course, the traceability time span is much longer than a week for the actual traceability demand in the carbon emission trading market.

## 5. Conclusions

To solve the problems faced by the implementation of carbon trading and low-carbon economy, and meet the needs of users at all levels for carbon footprint traceability, this paper further improves and perfects the distributed traceability concept of “traceability off the chain and verification on the chain“, constructs an interactive traceability structure that supports type filtering, and forms an efficient and a credible traceability system for various enterprise’s and product’s carbon footprint. It provides a practical solution for implementing blockchain technology into more difficult application domains. The simulation results show that the carbon footprint traceability system proposed in this paper, as well as the interactive traceability structure supporting type filtering and the corresponding traceability process, can achieve efficient traceability and tracking of carbon emission-related data on the transaction chain. In addition to easing the strain on full nodes to retrieve data, it also significantly increases traceability’s effectiveness, especially for long-span transaction chains. Since the distributed traceability concept is adopted, the traceability pressure of the full node is greatly reduced to liberate the computing power of the full node and shift the focus of work to verification, supervision, and other more valuable fields.

Conceptually speaking, we further divide the traceability process into three stages: locating, retrieving, and verifying. On the distributed storage structure, we build a three-layer structure to realize the independence of locating functions. With respect to the algorithm, it supports type filtering while ensuring efficiency. Compared with the previous concept of “traceability off the chain and verification on the chain”, this research further divides the off-chain traceability process into two steps (locating and retrieving), and finally divides the distributed traceability process into three stages: locating, retrieving, and verifying. Furthermore, the original two-layer interactive traceability structure is improved, and a three-layer interactive traceability structure is formed, which is divided into a verification layer, location layer, and storage layer. At the same time, based on the three-layer structure, a new traceability algorithm is designed, which makes the location process relatively independent. In this paper, the locating process is separated from the retrieving and verifying process instead of interleaving these processes, which allows us to perform limited and filtered data retrieval and verification of data after locating. This improvement can not only improve the efficiency but also reduce network congestion caused by large amount of data and provides a more feasible and flexible scheme for accurate and efficient traceability.

However, this study is not without limitations. Owing to the complexity of the file structure and the addition of Pointer files, the whole traceability process is more dependent on the interaction between users and the IPFS system. As a result, the network state worsens, which has a significant impact on the overall traceability efficiency. The traceability efficiency is highly connected to the frequency of interactions. Additionally, too many exchanges will significantly reduce the efficiency of traceability. To lessen the impact of network state on the effectiveness of traceability, we will concentrate on finding a solution to the issue that interaction frequency may be too high in the future.

## Figures and Tables

**Table 1:** An example of a pointer file.

Key	Value
Pre.BlockHeight	846
Pre.TxID	“1ecf8e326fb026229faf09584713d2aad8e1dca87e122f6563de09a6c68b0cf0”
Pre.CID	“QmWfaMJPv26p7JS2x7HW1UMPXUW61ySrn5n9SWur6Y47vb”
Category	[N, N, N, N, N, N, N, N, N, N,]
CIDs	[“QmVDP1uapHmbfYbW4VACYc6qEMWJb5vUDtPYoTidF417ma”, “QmeVb3baUtzMtRKgqxjUDJkCkNDejWSFiVph83Q2qEPMpT”, “QmV75Kypk5MM72SbdYbvj5gePNpHnx79QbNMj5wcB522ad”, “QmUGCyJMyFchEAcfcCYx48C562TP96TBWKRVKtS7kkU5pv”, “QmSakTYrwNZdGpupT54zVxfK5rJtSLQR7KQkM9DKUk52WP”, “QmTjWpJVj6c8rkPdwmhz4PjqhQMWhwBJvmDXb3pha8UU28”, “QmPpLSAp3YuNk7ezod5nPrDhCmLaWhopmGARygUJHVGDgJ”, “QmNT3NZFpmzHADLaxWUKGevHutdcNxFn31gSqjBRZoTmr3”, “QmYYX8TA6p3sjQxwtc7c9C8afUSMPHoSWrp1iKvmhkgm2c”, “QmbogYtv44bG8ySuxPM8UhccRpj1PFqJX6rqULtnob3LNB”]
Chain level	4

## Data Availability

The data used to support the findings of this study are available from the corresponding author upon request.
